# Caffeic Acid Phenethyl Ester Inhibits Epithelial-Mesenchymal Transition of Human Pancreatic Cancer Cells

**DOI:** 10.1155/2013/270906

**Published:** 2013-04-04

**Authors:** Ming-Jen Chen, Shou-Chuan Shih, Horng-Yuan Wang, Ching-Chung Lin, Chia-Yuan Liu, Tsang-En Wang, Cheng-Hsin Chu, Yu-Jen Chen

**Affiliations:** ^1^Division of Gastroenterology, Department of Internal Medicine, Mackay Memorial Hospital, Taiwan; ^2^Mackay Medicine, Nursing and Management College, Taipei, Taiwan; ^3^Department of Radiation Oncology, Mackay Memorial Hospital, No. 92, Sec. 2, Chungshan North Road, Taipei, Taiwan

## Abstract

*Background*. This study aimed to investigate the effect of propolis component caffeic acid phenethyl ester (CAPE) on epithelial-mesenchymal transition (EMT) of human pancreatic cancer cells and the molecular mechanisms underlying these effects. *Methods*. The transforming growth factor *β* (TGF-*β*-) induced EMT in human pancreatic PANC-1 cancer cells was characterized by observation of morphology and the expression of E-cadherin and vimentin by western blotting. The migration potential was estimated with wound closure assay. The expression of transcriptional factors was measured by quantitative RT-PCR and immunocytochemistry staining. The orthotopic pancreatic cancer xenograft model was used for *in vivo* assessment. *Results*. The overexpression of vimentin was attenuated by CAPE, and the alteration in morphology from polygonal to spindle shape was partially reversed by CAPE. Furthermore, CAPE delayed the TGF-*β*-stimulated migration potential. CAPE treatment did not reduce the expression levels of Smad 2/3, Snail 1, and Zeb 1 but inhibited the expression of transcriptional factor Twist 2. By using an orthotopic pancreatic cancer model, CAPE suppressed the expression of Twist 2 and growth of PANC-1 xenografts without significant toxicity. *Conclusion*. CAPE could inhibit the orthotopic growth and EMT of pancreatic cancer PANC-1 cells accompanied by downregulation of vimentin and Twist 2 expression.

## 1. Introduction

Pancreatic cancer remains a major unsolved health problem and the fourth leading cause of cancer-related death in the US [1]. Late diagnosis, rapid progression, and resistance to chemo- and radiotherapy render the high mortality of pancreatic cancer. The 5-year survival rate for all stages of pancreatic cancer is only approximately 5% and is only 10–25% for those with locoregional disease even after curative surgery [2]. Although gemcitabine is currently the drug of choice for chemotherapy [3], its low objective response rate remains unsatisfactory [4, 5]. 

Epithelial to mesenchymal transition (EMT) is known as a key step during embryonic morphogenesis and is involved in the progression of primary tumors toward metastasis [6]. EMT is characterized by loss of epithelial cell polarity, loss of cell-cell contacts, and acquisition of mesenchymal markers to highly motile fibroblast-like or mesenchymal features including migration potential, invasiveness, and resistance to apoptosis [7, 8]. EMT of cancer cells also correlates with cancer stem cell characteristics such as chemotherapy resistance [9, 10]. For example, an increased expression of EMT and stem cell markers in drug-resistant pancreatic cancer cells has been reported [11, 12]. 

Loss of E-cadherin expression and increasing vimentin expression are regarded as the important indicators of EMT initiation process [13]. Several cytokines are reported to induce EMT in pancreatic cancer cells, such as transforming growth factor *β* (TGF-*β*) on PANC-1 cells [14]. The transcriptional factors Snail and Twist 2 have been described to be direct repressors of E-cadherin *in vitro* and *in vivo* [15–17]. New therapeutic agents as EMT signaling inhibitors are therefore expected to overcome the metastasis, invasiveness, or drug resistance [18, 19].

Propolis is a wax-like resinous substance collected by honeybees from tree buds or other botanical sources and used as cement to seal cracks and support the architecture of beehives. It has been a popular folk medicine through the age and claimed with beneficial effect on human health. Caffeic acid phenethyl ester (CAPE), a naturally occurring compound isolated from the extract of propolis with well-known antioxidant activity [20], has been reported to have anti-inflammatory properties involving the inhibition of certain enzyme activities such as xanthine oxidase and cyclooxygenase and transcriptional factor NF-*κ*B activation [21–23]. Our previous work showed that CAPE quickly entered HL-60 cells and caused glutathione depletion [24], mitochondrial dysfunction, and caspase-3 activation [25]. It could inhibit the growth of human pancreatic cancer PANC-1 and BxPC-3 cells involving activation of caspase-3 and -7 and perturbation of the mitochondrial transmembrane potential to induce apoptosis. *In vivo*, intraperitoneal injection of CAPE (10 mg/kg/day) to BALB/c mice reduced the pulmonary metastatic capacity of CT26 cells in association with a decreased plasma VEGF level [26]. 

In the present study, we evaluated the effect of CAPE on EMT of human pancreatic cancer cells as well as the tumor growth *in vivo*.

## 2. Methods

### 2.1. Cell Lines and Culture Conditions

The human pancreatic cancer PANC-1 cells which were derived from a female cancer patient with K-ras and p53 mutation were purchased from the American Type Culture Collection (ATCC, Rockville, MD, USA). PANC-1 cells were cultured in DMEM (Biosource, Camarillo, CA, USA) and supplemented with 10% heat-inactivated fetal bovine serum (Biological Industries, Israel) at 37°C in a humidified 5% CO_2_ incubator. The cells were passaged every 2 to 3 days with TEG solution (0.25% trypsin, 0.1% EDTA, and 0.05% glucose in Hanks' balanced salt solution) and maintained in exponential growth. 

### 2.2. Reagents and Treatment

CAPE was purchased from Sigma Chemical Co. (St. Louis, MO, USA) and was dissolved in DMSO. The PANC-1 cells were cultured in a 96-well microplate for 18 h at an initial concentration of 5 × 10^5^/mL and grown at 37°C in a humidified 5% CO_2_ incubator. For induction of EMT, TGF-*β* 5 ng/mL (R&D Systems, Inc.) was added to the cells 2 h before CAPE (5 *μ*g/mL) treatment. PANC-1 cells, either untreated or pretreated with TGF-*β* and cotreated with CAPE and TGF-*β*, were harvested at various times from 24 h to 72 h.

### 2.3. Assessment of Cell Viability and Cell Morphology

The numbers of viable cells were estimated by using a trypan blue dye exclusion test. After various treatments, cells were collected to examine the morphology under an Olympus light microscope at a magnification of 1000x.

### 2.4. Wound Closure Assay

The wound closure assay was performed to examine the migration potential of pancreatic cancer cells. Briefly, pancreatic cancer cells were grown to full confluency in silicone inserts (Grid-500, ibidi GmbH, Germany) with a defined 500 *μ*m cell-free gap and incubated in complete medium. The wound gap was observed by phase microscopy. All experiments were repeated and triplicated. 

### 2.5. Western Blotting

Whole-cell lysates were prepared from cells treated at days 1, 2, and 3. The membrane was blocked with 5% defatted milk and then immunoblotted with primary antibodies including E-cadherin, vimentin, Smad 2/3, and phosphorylated Smad 2/3 (BD Transduction Laboratories) at room temperature for 2 hours. This was followed by addition of horseradish peroxidase-labeled secondary antibodies (Chemicon, Single Oak Drive, Temecula, CA, USA) and developed using the enhanced chemiluminescence system (Amersham Pharmacia, Piscataway, NJ, USA). The expression of *β*-actin was used as an internal control. 

### 2.6. Real-Time PCR Expression of Snail 1 on PANC-1 Cell Line

Total RNA was isolated from PANC-1 cells and purified using RNeasy Mini Kit (Qiagen), supplemented with RNase-free DNase (Qiagen). cDNA was obtained using the iScript Select cDNA Synthesis Kit (Bio-Rad Laboratories AB), and the absence of DNA contamination was verified by excluding reverse transcriptase. cDNA aliquots were subjected to PCR reactions using the QuantiTect SYBR Green PCR Kit (Qiagen) to amplify Snail 1 and GAPDH with primers using QuantiTect primer assays (Qiagen). PCR reaction was carried out as follows: 15 min at 95°C, 15 s at 94°C, 30 s at 55°C, and 30 s at 72°C. Each cycle was repeated for 40 times according to the manufacturer's recommendations by using the Rotorgene RG-3000A thermal cycler and Rotorgene 6.0 software (Corbett Research). On the basis of the comparative Ct method, gene expression levels were calculated and that of untreated cells was used as a control.

### 2.7. Immunocytochemistry Staining of Twist 2, Zeb-1 on PANC-1 Cells

For immunocytochemistry staining analysis of Twist 2 and Zeb 1, cells were incubated with the anti-Twist 2 and Zeb 1 antibodies (Abcam, Cambridge, MA, USA) overnight at 4°C. The proportion of cells with Twist 2 and Zeb 1 staining in cell nucleus was calculated at a high-power field for 10 different portions on microscopy.

### 2.8. Orthotopic Implantation of Xenografts

Male BALB/c nude mice, between 6 and 8 weeks old, were used in accordance with institutional guidelines. PANC-1 cells were harvested at a concentration of 5 × 10^6^/mL from subconfluent cultures. Tumor was generated by direct orthotopic injection of PANC-1 cells into the pancreatic tail. To prevent leakage, a cotton swab was gently held for 1 min over the site of injection. The abdominal wound was then closed with sutures. Thirty mice with confirmed tumor growth at day 10 were randomized into 3 groups with a similar average body weight in each group. Group A (*n* = 10) was treated with DMSO intraperitoneally as vehicle control. Group B (*n* = 10) was treated with CAPE at 10 mg/kg three times a week for a total of 20 doses intraperitoneally. Group C (*n* = 10) was treated with gemcitabine 50 mg/kg every week for 7 doses intraperitoneally. The treatment was continued for 6 weeks, at which half the mice in the three groups (*n* = 5 for each) were sacrificed and necropsied at the 53rd day, and the remaining mice were sacrificed and necropsied at day 90. Tumors were excised and the tumor size was measured as (1/2)*ab* ^2^ (*a* = the maximal diameter and *b* = the minimal diameter). Before necropsy, blood samples were collected for measurement of white blood counts every week in all groups of mice. 

### 2.9. Immunohistochemistry Staining of Twist 2 in PANC-1 Xenograft

For immunohistochemical analyses, excised tumors were fixed in formalin and embedded in paraffin. Antigen was retrieved using target retrieval solution (pH 9.0) (Dako). Primary anti-Twist 2 (Abcam) was incubated and was detected using the MM-HRP-Polymer Kit (Biocare Medical). An oncologist with pathological expertise blinded to grouping of specimens examined the stained slides to estimate the expression level of Twist 2 in a semiquantitative manner. The proportion of cells with Twist 2 and Zeb 1 staining in cell nucleus was calculated for more than 200 cells at high-power field in 10 different portions on microscopy.

### 2.10. Statistical Analysis

Data are presented as means ± standard error of mean (SEM). Significance between means was assessed by analysis of variance (ANOVA) followed by Fisher's test or the Wilcoxon signed-ranks test for multiple comparisons. *P* < 0.05 was considered significant.

## 3. Results

### 3.1. Effect of CAPE Treatment on TGF-*β*-Induced EMT in PANC-1 Cells

**Figure 1:**
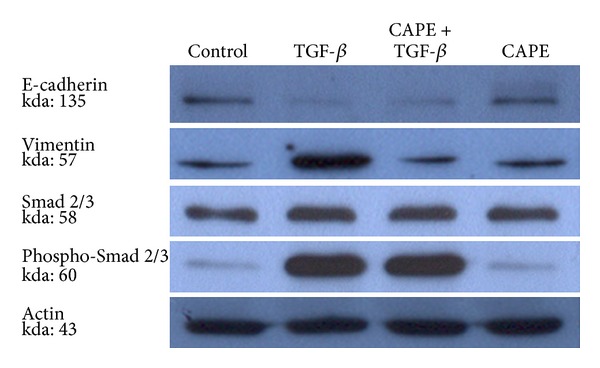
Effect of CAPE treatment on EMT markers expression. PANC-1 cells exhibited a weak expression of E-cadherin and strong expression of vimentin by TGF-*β* stimulation. The downregulation of E-cadherin expression and upregulation of vimentin expression, markers of EMT, were reversed by CAPE treatment, but CAPE treatment did not reduce the expression levels of Smad 2/3 (at 24 h).

**Figure 2:**
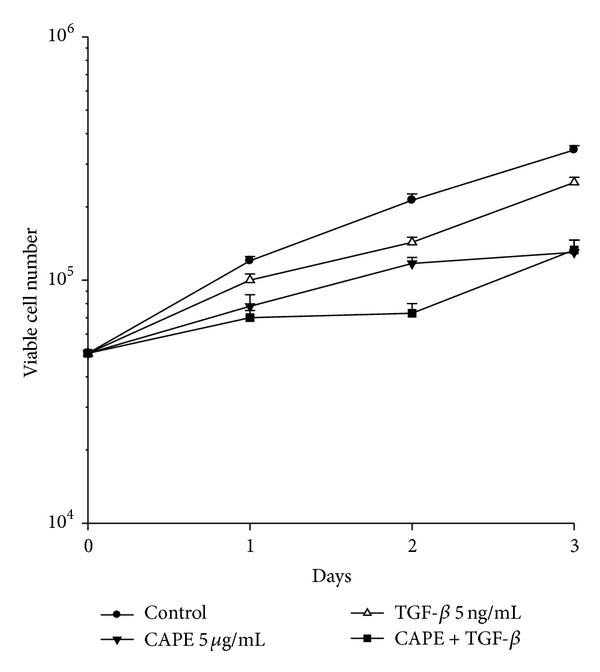
Assessment of cell viability. For induction of EMT, TGF-*β* (5 ng/mL) was added to the cells 2 h before CAPE (5 *μ*g/mL) treatment. PANC-1 cells, either untreated or pretreated with TGF-*β* and cotreated with CAPE and TGF-*β*, were harvested at various times from 24 h to 72 h. CAPE treatment reduced the viability of TGF-*β*-stimulated cells.

**Figure 3:**
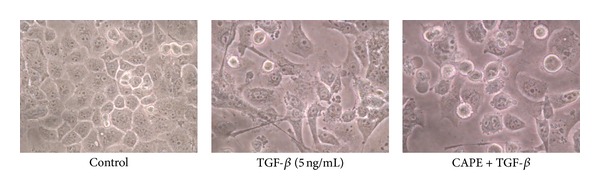
Assessment of cell morphology. TGF-*β* triggered PANC-1 cells from polygonal to spindle shape with abundant cell-cell bridging, and this feature was reversed by CAPE addition at 72 h.

**Figure 4:**
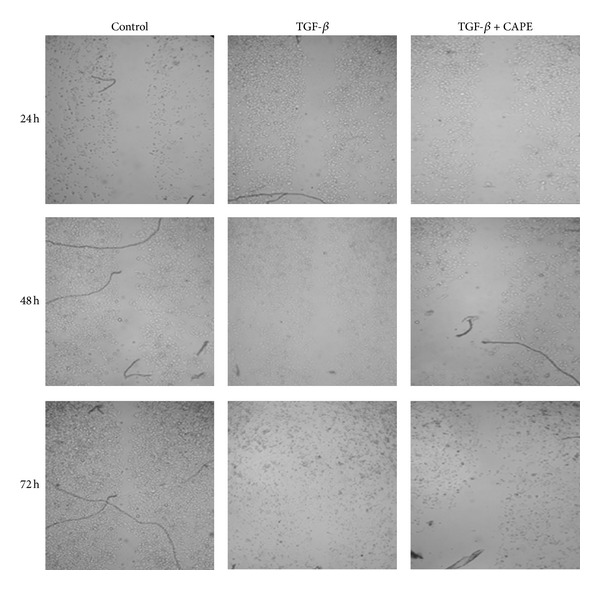
The wound closure assay for the migration potential. Migration of PANC-1 cells, a hall marker of EMT for invasiveness, was augmented by TGF-*β*, and it could be delayed by CAPE treatment under 72 h observation.

By TGF-*β* stimulation, pancreatic cancer PANC-1 cells exhibited a transition from epithelial to mesenchymal characteristics. The downregulation of E-cadherin expression and upregulation of vimentin expression, markers of EMT, were reversed by CAPE treatment (Figure 1). CAPE treatment reduced the viability of TGF-*β*-stimulated cells (Figure 2). As for morphological alteration, TGF-*β* triggered PANC-1 cells from polygonal to spindle shape with abundant cell-cell bridging, and this feature was reversed by CAPE addition (Figure 3). Migration of PANC-1 cells, a hall marker of EMT for invasiveness, was augmented by TGF-*β*, and it could be delayed by CAPE treatment under 72 h observation (Figure 4).

### 3.2. Expression of Signaling Molecules Related to EMT

**Figure 5:**
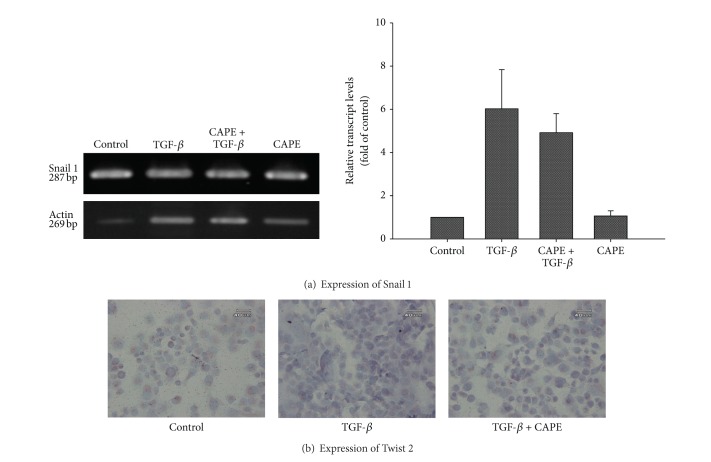
Expression of signaling molecules related to EMT. By real-time PCR expression, Snail 1 was upregulated by TGF-*β*, but it was not affected by CAPE treatment. By immunocytochemistry stain, the nuclear expression of Twist 2 was enhanced by TGF-*β* (44%), and this effect could be reversed by CAPE (12%).

At effective condition of TGF-*β* treatment to trigger EMT, the expression of Smad 2/3 and its phosphorylated form was increased, indicating the existence of TGF-*β* signaling. However, the upregulation of Smad 2/3 was not altered by CAPE treatment (Figure 1). To further elucidate the mechanism of action, we examined the expression of transcriptional factors. As demonstrated in Figure 5(a), Snail 1 was upregulated by TGF-*β*, but it was not affected by CAPE treatment (858.0 ± 1434.6 versus 30.6 ± 29.1, *P* = 0.45; by comparative Ct method). By immunocytochemistry stain, we found that nuclear expression of Twist 2 was enhanced by TGF-*β*, and this effect could be reversed by CAPE (Figure 5(b)), indicating a putative target of CAPE on PANC-1 cells for EMT modulation. 

### 3.3. Orthotopic Pancreatic Cancer PANC-1 Xenograft

**Figure 6:**
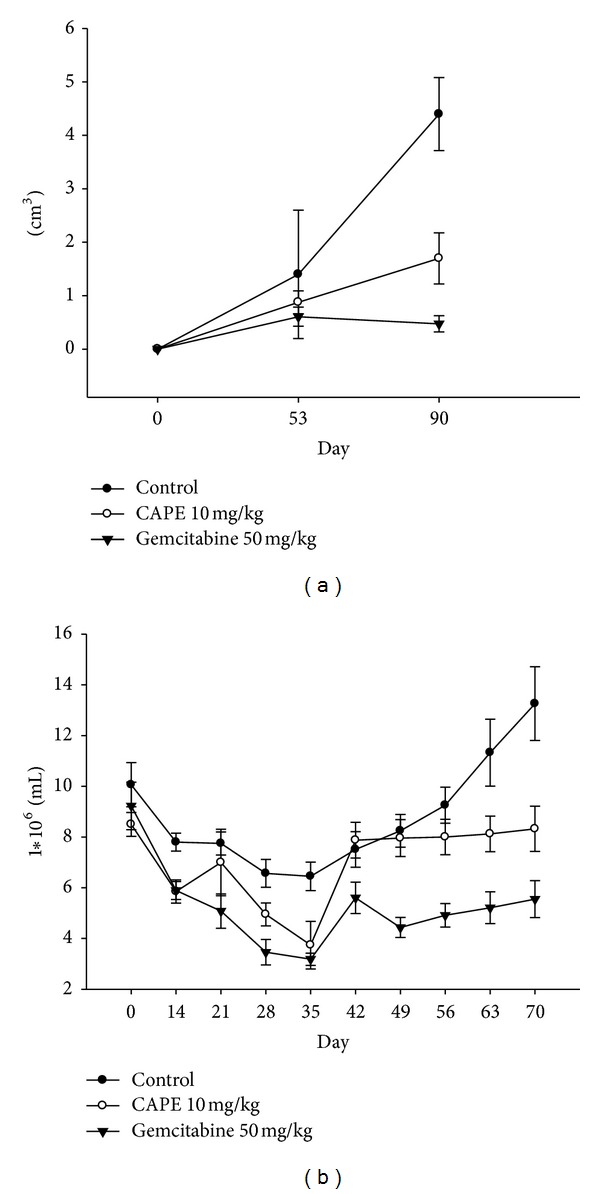
Orthotopic pancreatic cancer PANC-1 xenograft. (a) At day 53 and 90, the volumes of the pancreatic tumor were suppressed in the CAPE-treated group although not as effective as gemcitabine. (b) There was a less bone marrow suppression in the CAPE-treated group than the gemcitabine-treated group during the treatment course by serial estimation of WBC counts.

All mice tolerated the treatment well. At day 53, the volumes of the pancreatic tumor were 1.4 ± 1.2 cm^3^ in the controls, 0.9 ± 1.2 cm^3^ in the CAPE-treated group, and 0.6 ± 0.2 cm^3^ in the gemcitabine-treated group (Figure 6(a)). At the 90th day, the volumes of the pancreatic tumor were 4.4 ± 0.7 cm^3^ in the control mice, 1.7 ± 0.5 cm^3^ in the CAPE-treated group, and 0.5 ± 0.2 cm^3^ in the gemcitabine-treated group (Figure 6(a)). There was a less bone marrow suppression in the CAPE-treated group than the gemcitabine-treated group during the treatment course by serial estimation of WBC counts (Figure 6(b)).

### 3.4. Validation of CAPE Effect on Twist 2 Expression *In Vivo*

**Figure 7:**
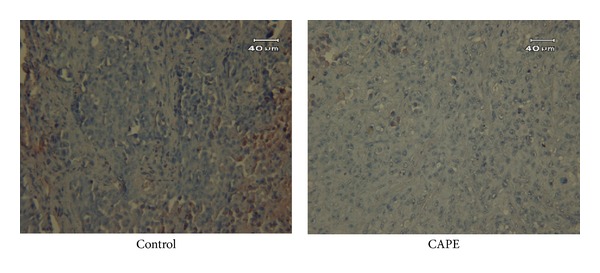
Immunohistochemistry staining of Twist 2 in PANC-1 xenograft. By immunohistochemistry stain, the expression of Twist 2 in PANC-1 xenograft was significantly suppressed by CAPE treatment. Extensive tumor necrosis with scanty cell-cell bridging by CAPE treatment was also noted.

By immunohistochemistry stain, we found that nuclear expression of Twist 2 but not Zeb 1 was enhanced by TGF-*β*, and this effect could be reversed by CAPE from 34% to 12% (Figure 7). Moreover, extensive tumor necrosis with scanty cell-cell bridging by CAPE treatment was also noted similar to that *in vitro* assay.

## 4. Discussion

Bioactive components from the propolis have been extensively explored to possess anticancer activity. However, in clinical practice, the treatment resistance and highly metastatic potential of pancreatic cancer remain the major challenge for oncologists. EMT has been regarded as a critical mechanism resulting in these unfavorable clinical features. Under this concept and based on previous anticancer investigations, we proposed that CAPE might have potential to modulate EMT in pancreatic cancer. The results demonstrated that CAPE could suppress EMT of PANC-1 cells with involvement of Twist 2 modulation.

E-cadherin is required for the formation of stable adherent junctions and thus the maintenance of an epithelial phenotype. Loss of E-cadherin expression is emerging as the most common indicator of EMT onset, and reduced expression of E-cadherin has been reported in various cancers, being associated with tumor progression and metastasis [27]. We examined the effects of TGF-*β* on the expression of EMT-related markers in the PANC-1 cells. As for results, TGF-*β* treatment reduced the expression of the epithelial marker E-cadherin but increased the expression of the mesenchymal marker vimentin. Treatment with CAPE slightly restored the expression of E-cadherin and markedly reversed the TGF-*β*-induced overexpression of vimentin at 24 h. It implicates that CAPE could suppress the EMT in pancreatic cancer. 

TGF-*β* may induce EMT through multiple distinct signaling mechanisms, including direct phosphorylation by ligand-activated receptors of transcription factors such as Snail 1 or Smad [28, 29]. In our study, we found TGF-*β*-induced overexpression of Smad 2/3 and Snail 1 in PANC-1 cells, but CAPE could not overcome this effect. Next, we postulated that CAPE might act through pathways other than Smad-inducing signaling during progression of EMT. 

Twist 2 has been known to cooperatively repress E-cadherin, leading to the induction of EMT in cancer cells. We found an inverse correlation between expressions of E-cadherin and Twist 2 in PANC-1 cells. However, the expression of Zeb 1 in nucleus was not significantly changed. It implicates that Twist 2 might be the target for CAPE effect on EMT. The further investigation for the causal relationship is needed.

*In vivo*, we found that CAPE, although not as effective as gemcitabine, is not significantly toxic while suppressing tumor growth. For cancer treatment with cytotoxic agents, the major dose limiting factor is their toxicity to normal cells and tissues. This safety consideration is particularly critical in the cancer patients. In this study, the concentration CAPE (5 *μ*g/mL) for inducing EMT was relatively low. In concentrations similar to those used in our study, CAPE has been reported to have selective cytotoxicity for cancer cells, to some extent sparing human umbilical vein epithelial cells, lung fibroblast WI-38 cells [30], and buccal mucosa fibroblasts [31]. Cytotoxic agents such as gemcitabine or 5-fluorouracil, for example, are myelosuppressive and thus prone to cause life-threatening neutropenia, anemia, or thrombocytopenia. CAPE does not seem as toxic as gemcitabine to bone marrow function. Novel therapeutic combinations using cytotoxic agents and/or EMT signaling inhibitors are therefore expected to circumvent the chemotherapeutic resistance of cancers characterized by sustained EMT signatures to achieve improvement on currently available chemotherapy. 

## Abbreviations

CAPE: Caffeic acid phenethyl ester
EMT: Epithelial-mesenchymal transition
TGF-*β*: Transforming growth factor *β*.
